# Recommendations and Protocols for the Safe Use of Tourniquets in Upper- and Lower-Extremity Surgeries

**DOI:** 10.1055/s-0045-1809689

**Published:** 2025-07-29

**Authors:** Antonio Tufi Neder Filho, Túlio Vinícius de Oliveira Campos

**Affiliations:** 1 Locomotor System Department, Faculdade de Medicina, Universidade Federal de Minas Gerais, Belo Horizonte, MG, Brazil; 2 Rede Mater Dei de Saúde, Belo Horizonte, MG, Brasil

**Keywords:** complications, lower extremity, protocols, tourniquets, upper extremity, complicações, extremidade inferior, extremidade superior, protocolo, torniquetes

## Abstract

The tourniquet (TNQ) interrupts blood flow to a given anatomical segment and has critical applications in orthopedic surgeries by providing a blood-free operating field. The risks and complications attributed to its use are increased pain, reperfusion injury, edema, deep venous thrombosis, and peripheral nerve injury. The main recommendations for TNQ use and to reduce the occurrence of complications include adequate limb padding; TNQ inflation to pressures of 50 mmHg and 100 mmHg above the perfusion pressure for the upper and lower limbs respectively; avoid TNQ use in children and patients with cachexia, lupus, and coagulopathy; avoid keeping the device inflated for more than 2 hours; and have a trained team alert to deflation, which is characterized by the possibility of bleeding, pulmonary embolism, and myonephropathic metabolic syndrome. The present update article summarizes the best evidence on TNQ use in orthopedic surgeries and proposes a protocol for its safe use.

## Resumo

O torniquete (TNQ) interrompe o fluxo sanguíneo para um determinado segmento anatômico e tem aplicação importante nas cirurgias ortopédicas ao proporcionar um campo operatório limpo de sangue. Os riscos e complicações atribuídos ao seu uso são aumento da dor, lesão por reperfusão, edema, trombose venosa profunda e lesão de nervos periféricos. As principais recomendações para se utilizar o TNQ e diminuir a ocorrência de complicações são: acolchoar adequadamente o membro; insuflar o torniquete até pressão 50 mmHg acima da pressão de perfusão para os membros superiores e 100 mmHg para os membros inferiores; evitar o uso em pacientes com caquexia, crianças, portadores de lúpus e coagulopatia; evitar manter o dispositivo insuflado por período superior a 2 horas; e ter equipe treinada e atenta para a desinsuflação, caracterizada pela possibilidade de sangramento, embolia pulmonar e síndrome metabólica mionefropática. Este artigo de atualização resume a melhor evidência acerca do emprego do TNQ nas cirurgias ortopédicas e propõe um protocolo para sua utilização segura.

## Introduction

The tourniquet (TNQ) is a device that interrupts blood flow to a specific anatomical segment. It can be used to provide a blood-free surgical field, to enable segmental anesthesia through the Bier technique, to facilitate puncture by increasing venous engorgement in the limb, and to control bleeding in severe extremity trauma. 1 2

Some studies 3 report TNQ-related risks, such as increased pain, reperfusion injury, edema, deep venous thrombosis (DVT), pain around the edges of the surgical wound, and peripheral nerve injury.

The knowledge of surgeons and of the team of assistants about the technique for TNQ placement, contraindications, and maximum time of use in orthopedic surgeries is often inadequate. 4

The current article aims to present an update on TNQ use in orthopedic surgeries and to propose a protocol based on these findings for its safe use.

## Tourniquet use on the upper limbs

The inflation pressures are lower and the time the TNQ remains in place is shorter in upper-limb surgeries compared with lower-limb surgeries. The maximum pressure is of 250 mmHg, and the average procedure time is of 30 minutes. The cuff can be on the arm or forearm. The latter positioning can cause the fingers to flex towards the palmar region and hinder the view of the surgical field. 2

**Fig. 1:**
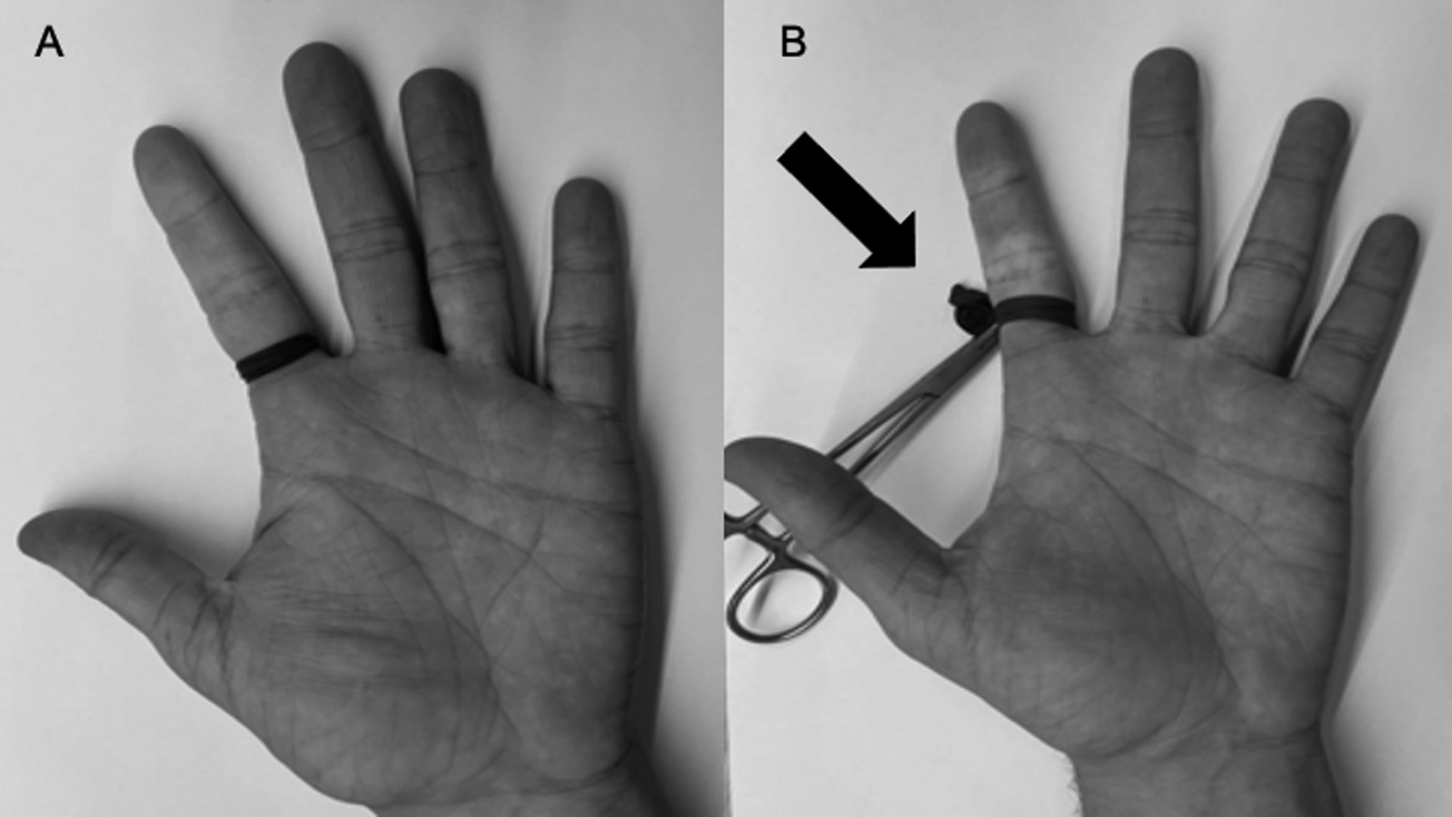
( **A** ) Elastic tourniquet applied to the base of the second finger. This type of device has no pressure control, and it should be colored to prevent forgetfulness. ( **B** ) Finger cot applied to the base of the finger. The use of a clamp makes it difficult to forget it, which could cause an iatrogenic injury to the extremity (black arrow).

The capillary pressure in the upper limbs is lower than in the lower limbs. Therefore, the pressure required to inflate the TNQ is lower in the upper limbs. 5 The fingers require a specific TNQ type for the upper limbs, using colored elastic bands or a finger cot at the base of the finger. The disadvantage of this type of TNQ is the inability to control the pressure. The recommendation is to hold it at the base of the finger with a clamp to avoid forgetting the segment and causing an iatrogenic amputation ( Fig. 1 ). 2

Comparative studies 2 have shown no differences among several methods for upper-limb exsanguination, such as limb elevation, using an Esmarch elastic band, or manual drainage. The outcomes evaluated were the ability to provide a clean field and hemodynamic repercussions. The comparison between silicone and pneumatic TNQs did not yield conclusive results. Some authors state that the pressure gradient at the limits of the TNQ is smaller in silicone devices, automatically exsanguinating the limb and presenting more rounded edges. 2

Tourniquet-related nerve injuries are 2.5 times more frequent in the upper limbs, and the radial nerve is the most affected. This increased risk may result from the reduced soft tissue coverage in this region. 2

## Tourniquet use on the lower limbs

There is controversy regarding TNQ use in lower-limb surgeries. The main arguments favoring these devices are the practicality of operating in a clean field and the reduced intraoperative blood loss. The negative points include a 1-point increase in the value attributed to pain on the visual analog scale (VAS), a 3-mg increase in opioid consumption on the first day after surgery, and transient loss of strength in the segment of TNQ application. 2

In a systematic review and meta-analysis, Zhang et al. 6 evaluated TNQ use in arthroscopic surgeries of the lower limbs; the authors did not identify differences in the surgical field visualization quality nor in operative time. Furthermore, they 6 highlighted that TNQ use may result in postoperative muscle weakness, and it modifies intraoperative parameters, such as patellar mobility in femoropatellar stabilization procedures. In a systematic review and meta-analysis including patients undergoing simple arthroscopy and ligament reconstructions, Wang et al. 7 evaluated 16 studies with 1,132 participants and concluded that the group without TNQ experienced lower levels of postoperative blood loss and lower intake of analgesic medication. These authors identified no differences in terms of surgical visibility pain, quadriceps strength, or operative time.

In anterior cruciate ligament reconstruction surgery, TNQ use resulted in worse quadriceps atrophy, weakness, and electroneuromyographic changes in the first 4 and 12 weeks postoperatively. There was no difference 1 year after surgery. 8 9

The use of TNQ in total knee arthroplasty is one of the most discussed topics in the recent literature. The studies debate the benefits and risks of TNQ use, the inflation/deflation timing, as well as the short- and long-term effects. The analysis of the studies should consider when they were conducted. Tranexamic acid, uncemented prostheses, and robotics in joint-replacement surgeries interfered with critical outcomes, such as blood loss. Therefore, assessing the context of arthroplasty performance before comparing studies is crucial.

In a systematic review and meta-analysis, Zhang et al. 10 compared TNQ release before and after wound closure in knee arthroplasties. The authors 10 included 1,010 patients and found no significant differences in terms of calculated blood loss, postoperative blood loss, hemoglobin drop, transfusion rate, major complications, or DVT. The group of TNQ release before wound closure presented a higher volume of total blood loss and longer operative time. However, releasing the TNQ before wound closure can reduce minor complications. 10

Han et al. 3 published a systematic review and meta-analysis with 29 randomized clinical trials and 2,512 knee arthroplasties; they concluded that TNQ decreased intraoperative blood loss and operative time and increased interdigitation of the cement mantle. They did not detect differences in terms of total blood loss volume, blood transfusion rate, or DVT rate. In contrast, TNQ use increased postoperative pain, edema, and length of hospital stay, and it worsened range of motion and functional scores. 3 Lu et al. 11 also found greater penetration of the cement mantle and highlighted the importance of this finding in implant longevity. The authors 11 reported no difference in blood loss but a higher occurrence of pain in patients using TNQs.

In a meta-analysis, Sun et al. 12 evaluated 14 randomized clinical trials with 1,720 total knee arthroplasties performed with tranexamic acid; they compared patients using TNQ or not during the procedure, concluding the device was correlated with lower levels of intraoperative blood loss and shorter operative time, but also, with higher levels of occult blood loss and decreased range of motion. None of the other parameters were different between the groups: hemoglobin, total blood loss, transfusion rate, drainage volume, VAS on the day of the surgery and 1, 2, 3, 5, 7, and 30 days after it, Hospital for Special Surgery (HSS) score at 7 days, 1 month, 3 months and 6 months after surgery, knee circumference, length of hospital stay, and complications, such as DVT and infection. 12 He et al. 13 compared knee arthroplasty patients using TNQ throughout the surgical procedure with those in whom the TNQ was activated only at the cementation time. The authors 13 concluded that the restricted TNQ use, only at cementation, decreased the drain volume in the postoperative period and increased the total blood loss without interfering with the blood transfusion rate; thus, they recommended the full-time use of TNQ.

In a Cochrane review, Ahmed et al. 14 concluded that TNQ increased the incidence of adverse events in total knee arthroplasty (relative risk: 1.73) and increased pain on the first postoperative day. Regarding the latter finding, the difference was of a single point on the VAS, so it was deemed irrelevant in the practical scenario. The authors 14 recommended that, in the case of TNQ use, the surgical team should inform the patient about the increased risk of complications.

Horlocker et al. 15 evaluated patients undergoing revision total knee arthroplasty with TNQ (mean operative time: 145 minutes). The rate of tibial and/or fibular nerve palsy was of 7.7%. Recovery was complete in 100% of the cases of tibial nerve and in 89% of the cases of fibular nerve palsies. The authors 15 evaluated the subgroup of procedures with TNQ time greater than 180 minutes: the incidence of neurological complications was lower in patients with a device deflation interval longer than 30 minutes (22%) compared with those with no interval (42%) or with an interval shorter than 30 minutes (39%).

Studies evaluating TNQ use in fracture treatment are scarce, and there are numerous questions regarding their methodology. In a systematic review of TNQ use in fracture treatment, Præstegaard et al., 16 did not identify TNQ-related benefits in trauma surgery.

## Risks and complications

Complications due to TNQ use may occur during the intraoperative and postoperative periods.

In the intraoperative period, the major concern is during TNQ pressure release. It is necessary to notify the team, who must know the most common complications. A potential large venous thrombus detaching from the limb requires adequate monitoring by the anesthesiology team. Although TNQ use is not an independent risk factor for thrombosis, the patients who often receive this device are at greater risk due to the nature of the procedure. Another complication that may occur at this time is myonephropathic metabolic syndrome, featuring metabolic acidosis, hyperkalemia, myoglobulinemia, myoglobinuria, and renal dysfunction. Paradoxically, TNQ release can increase the thrombolytic activity and activate antithrombin III and protein C pathways, potentially implicated in the increased bleeding shortly after TNQ release. 17

Most studies on TNQ muscle effects used animal models with pressures of approximately 350 mmHg for 2 hours. These studies 5 revealed two potential types of muscle injury: direct compression by the TNQ and distal ischemic injury. The highest effect on muscle strength occurred in the first days after use, with recovery of more than 80% of strength after 2 weeks. The authors 5 reported higher nerve resistance to acute injury. However, nerve injury recovery is slower than muscle injury recovery.

In addition, paresthesia and temporary or permanent paralysis may result from TNQ use in 0.024% of the cases, potentially from uncalibrated devices. Studies 1 show that 35% to 65% of the devices are not properly calibrated. Clinically, the incidences of fibular and tibial nerve injuries are higher when the TNQ remains inflated for more than 150 minutes. 15

Pulmonary embolism can occur during limb emptying. This complication is more common in trauma surgeries, but there are reports 1 of its occurrence in elective procedures. Therefore, it is crucial to inform the moment of TNQ release to all professionals assisting the patient during the perioperative period. 1

In children, TNQ increases the heart rate, blood pressure, and core body temperature by reducing the heat exchange area. Complications classically described for adults, such as pain, limb edema, compartment syndrome, skin pressure injuries, or chemical burns, have also been reported in children. Reilly et al. 18 used limb occlusion pressure as a parameter to define TNQ inflation in arthroscopic knee surgeries in children. The average pressure was of 151 mmHg. In this study, the authors 18 observed a group of patients submitted to a pressure arbitrarily set at 300 mmHg. There were no complications, and the TNQ effect on the surgical field visualization was adequate in both groups. The recommended TNQ inflation pressure in children is variable; some authors 18 19 have reported good surgical site visualization starting at 25 mmHg above the perfusion pressure for the upper limbs and 50 mmHg for the lower limbs.

**Fig. 2:**
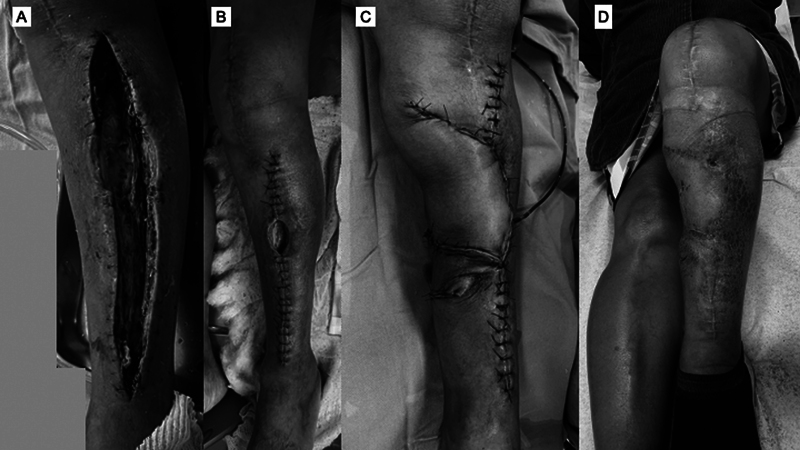
Patient with acute compartment syndrome in the immediate postoperative period of total knee arthroplasty. The tourniquet is one of the justifications for this complication. ( **A** ) Fasciotomy; ( **B** ) fasciotomy closure and closure defect in the anterolateral region of the leg; ( **C** ) myofasciocutaneous flap of the medial gastrocnemius for lesion closure; and ( **D** ) final appearance after 3 months of follow-up.

Arterial injuries occur mainly in patients with previous arterial disease, and they present clinically as thrombosis. Acute compartment syndrome is described and attributed to the use of TNQ for a prolonged period, to the application of very high pressure, and to the miscalibration of the device ( Fig. 2 ).

In a systematic review, Haladdin et al. 20 qualitatively evaluated the effect of anesthetic interventions, antioxidant use, and preconditioning on TNQ-related oxidative stress. All five studies assessing propofol and the three studies evaluating preconditioning demonstrated their beneficial effect on oxidative stress. The authors 20 emphasized the low quality of the existing evidence on the subject and the lack of studies on the clinical repercussions of the interventions, proposing the analysis of the clinical effect of these agents as a research line.

Table 1 summarizes the main differences in TNQ application on the upper and lower limbs.

**Table 1:** Configuration for the application and complications related to the use of upper- and lower-limb tourniquets

	Upper limb	Lower limb
**Maximum inflation pressure**	250 mmHg	300 mmHg
**Inflation pressure**	50 mmHg above the perfusion pressure	50 mmHg above the perfusion pressure
**Complications**	• Forgetfulness (fingers); and • Peripheral nerve injury (smaller amount of surrounding soft tissue)	• Thrombosis; and • Reperfusion syndrome

## Suggested protocol for tourniquet application

The following protocol organizes and summarizes the information collected from the papers 1 2 5 21 22 23 used to write the current update article. The key moments for TNQ application are: 1) limb preparation; 2) TNQ characteristics; 3) ischemia time; and 4) pressure.

## Limb preparation

Protect the skin below the TNQ with wool, cotton, or synthetic fabric bandages without exceeding two layers, since more layers reduce the TNQ effectiveness. 1

Take care to avoid accumulation of antiseptic solution, especially alcohol, under the TNQ, as it can result in skin lesions, especially in elderly subjects and children. Isolation with plastic or compresses can help to prevent this accumulation. 1

Before TNQ inflation, elevate the limb for approximately 2 minutes to remove the blood. Another option is to use compressive bandages. Inflate the TNQ quickly, since venous flow interruption occurs first, followed by interruption of the arterial flow. If the time between these milestones is long, venous pressure increases, and the TNQ effect may be paradoxical in bleeding. 1 2

Another critical detail is administering prophylactic antibiotic agents before cuff inflation, ideally before starting limb preparation, 1 hour before the skin incision. 5

## Tourniquet characteristics

The width of the cuff correlates with the effectiveness of the device. The ratio of the cuff width to the limb circumference is critical to define the occlusion pressure. Very narrow cuffs require higher inflation pressures to be effective. Authors 21 have identified that, if the ratio between the cuff width and the limb diameter is higher than 0.3:1, the pressure for perfusion occlusion is lower than the systolic pressure, potentially reducing the risk of injuries resulting from TNQ use.

**Fig. 3:**
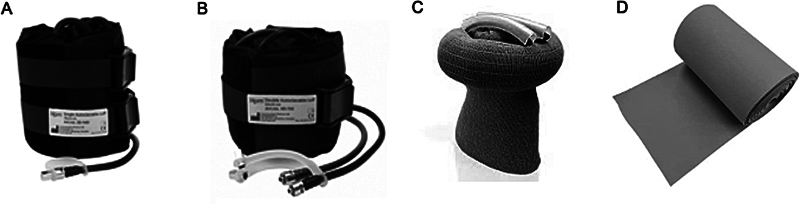
Types of tourniquets available. ( **A** ) Simple pneumatic tourniquet. ( **B** ) Double tourniquet, which enables the alternation of inflation points. ( **C** ) Sterile silicone tourniquet. ( **D** ) Esmarch bandage.

Most TNQs are pneumatic and non-sterile, and they can be used numerous times. However, some silicone devices are sterile and for single use. The advantage of these devices is the possibility of reaching difficult-to-access sites, such as the root of the thigh, and the fact that they enable the fixation of femoral diaphyseal fractures with retrograde nailing, for instance ( Fig. 3 ).

## Ischemia time

In subjects under 50 years of age, the 2-hour ischemia time limit is justified by the acidosis that occurs in the venous segment and its potential repercussions. Some authors have proposed the 30-minute interval after 2 hours of TNQ application to extend its use time. Cooling the limb also reduces the effects of ischemia and can be used in procedures expected to last more than 2 hours. For longer surgeries, the use of double cuffs enables the alternation of the compression area. 1

## Pressure

Regarding the maximum pressure to be applied, the authors of a review article 5 by the American Academy of Orthopaedic Surgeons (AAOS) recommends the use of a maximum pressure of 250 mmHg for upper-limb surgeries and of 300 mmHg for lower-limb surgeries.

Another parameter is the limb occlusion pressure, calculated with Doppler and defined as the pressure required to interrupt the blood flow to that anatomical segment. This process is recommended mainly for patients with severe arterial disease and calcification of the artery wall, in whom the pressure required to interrupt the arterial flow to the limb may be overestimated. 2

Ding et al., 22 in a meta-analysis published in 2019, concluded that defining the TNQ inflation pressure based on the systolic blood pressure measured with Doppler or pulse oximetry and the limb circumference reduces the cuff inflation value, improves the hemostatic effect, and decreases the incidence of complications.

Surgeons and anesthetists are very concerned about the time the TNQ remains in place. However, the pressure applied is a critical factor in this process. The pressure applied to the TNQ is related to the physical injury to the nodes of Ranvier, a significant factor in the occurrence of complications. 23

**Fig. 4:**
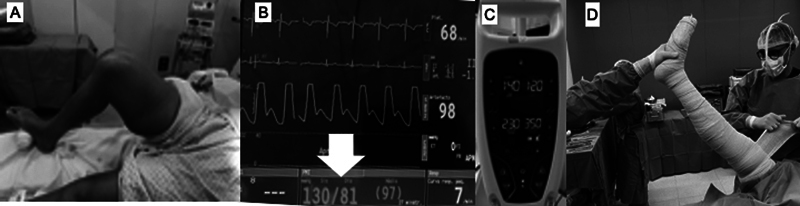
Main steps to observe when placing the tourniquet for orthopedic surgeries. ( **A** ) Cuff at the root of the limb with skin protection using crepe bandages. ( **B** ) Measurement of the patient's systolic blood pressure (white arrow). ( **C** ) Cuff inflation at 100 mmHg above the systolic pressure or the limb perfusion pressure. ( **D** ) Limb emptying with a compressive bandage before tourniquet inflation. After this sequence, the surgical team should record on the time-out table the time of cuff inflation, which should be maintained for a maximum period of 120 minutes.

Fig. 4 illustrates the most important steps to follow when applying TNQs in orthopedic surgeries: 1) TNQ positioning; 2) Definition of the inflation pressure; and 3) limb preparation.

**Table:** 

Key points for TNQ use in orthopedic surgeries
***Before TNQ inflation***
Elevate the limb to empty it.
Administer prophylactic antibiotics before inflation and 1 hour before the incision.
Place adequate padding under the cuff.
Set the inflation pressure: 50 mmHg above the perfusion pressure for the upper limbs and 100 mmHg for the lower limbs.
Be careful with special patients: children and patients with systemic lupus erythematosus, coagulopathy, and cachexia. Evaluate use on a case-by-case basis.
**After TNQ inflation**
Avoid ischemia for longer than 2 hours.
Have a trained support team to ensure compliance with inflation/deflation times: a maximum of 2 hours, followed by at least 30 minutes of reperfusion.
**Before TNQ deflation**
Inform the support team in the operating room, especially the anesthesiologist, to pay attention to acute repercussions: 1) pulmonary embolism; 2) myonephropathic metabolic syndrome; and 3) bleeding.

## Conclusion

The use of the TNQ makes the surgical procedure faster and safer, but all points discussed in this protocol must be observed to obtain its benefits and minimize its risks as much as possible.
